# Sick Appliances for the Household

**Published:** 1887-01-29

**Authors:** 


### Till the Doctor Comes.

[Inquiries and Communications for this column should be addressed, "Physician," care of the Editor of The Hospital.]

### IX.—SICK APPLIANCES FOR THE HOUSEHOLD.

**Medicine Measures.** One of these should be kept in every house: it is not safe to trust to ordinary spoons.

| | | |
|---|---|---|
| 1 teaspoonful | = | 1 drachm, or 60 drops. |
| 1 dessertspoonful | = | 3 drachms. |
| 1 tablespoonful | = | ½ an ounce, or 4 drachms. |
| 2 tablespoonfuls | = | 1 ounce, the ordinary dose for a grown-up person. |

Keep all poisons in blue *fluted* bottles; they can thus be recognised, even in the dark.

**Bed-Rests.** Messrs Hooper & Co., Pall Mall East, supply a useful and inexpensive bed-rest, made of galvanised iron or cane, which is strong, light and portable. To use this rest the bolster and pillows must be removed, and the rest firmly placed on the mattress. A pillow is then placed over the framework, against which the patient leans. This rest will be found to answer its purpose quite as well as the more complicated ones. A chair makes a very efficient bed-rest in an emergency. The legs of the chair are to be turned upwards and the seat backwards, so that the upper part of the back of the chair slopes gradually under the back of the patient, who rests against it on pillows.

*Drugs and Apparatus that may be usefully kept in any house.*

| | |
|---|---|
| Higginson's Enema Syringe. | Gutta-Percha Tissue. |
| Forceps, to remove splinters. | Cotton Wool. |
| Lancet. | Mackintosh. |
| Graduated Glass Measure. | Strapping. |
| Lint. | |

What is known as soap-strapping is preferable ; it is much less irritating than the ordinary yellow adhesive-strapping, and is the only sort that should be used to a wound.

| | |
|---|---|
| Epsom Salts. | Sal Volatile. |
| Castor Oil. | Sulphate of Zinc. |
| Zinc Ointment. | Linseed Meal. |
| Vaseline. | Poppy Heads. |
| Soap Liniment. | Bran. |
| Compound Camphor Liniment. | Alum. |
| | Lunar Caustic in Holder. |
| Ipecacuanha Wine. | Sweet Oil. |
| Turpentine. Laudanum. Strong Ammonia (Hartshorn). | For external use only. |
| Carbolic Acid (Liquid). Sanitas (Liquid and Powder). Condy's Fluid. Chloralum. Roll Sulphur. | Useful for disinfecting purposes. |

All are not absolutely necessary ; a selection may be made. Most of their uses will be indicated in future pages.

**To introduce Eye-drops into the Eye.** Take a quill pen and round off the point; dip it into the bottle containing the lotion, of which it will take up one or two drops. Then draw down the lower lid, and touch the inner red surface of the lid with the tip of the quill ; the drops will at once flow over the surface of the lid, which must then be released. A camel's hair-brush may be used in the same way. If there is very much discharge, as in the inflamed eyes of children, it is much better to lay the child on its back with the head level, and pour plenty of the lotion into the inner corner of the closed lids ; then open both the upper and lower lids, and the lotion will run over the eye, carrying all discharge away with it, and escaping at the outer angle of the eye.

(*To be continued.*)

